# Dried blood spots for *Streptococcus pneumoniae* and *Haemophilus influenzae* detection and serotyping among children < 5 years old in rural Mozambique

**DOI:** 10.1186/s12887-020-02209-3

**Published:** 2020-07-02

**Authors:** Fabiana C. Pimenta, Benild Moiane, Fernanda C. Lessa, Anne-Kathryn L. Venero, Iaci Moura, Shanda Larson, Sergio Massora, Alberto Chaúque, Nelson Tembe, Helio Mucavele, Jennifer R. Verani, Cynthia G. Whitney, Betuel Sigaúque, Maria G. S. Carvalho

**Affiliations:** 1 Division of Bacterial Diseases, National Center for Immunization and Respiratory Diseases, Centers for Disease Control and Prevention, Atlanta, 30329 USA; 2 Centro de Investigação em Saúde de Manhiça, 1929 Maputo, Mozambique; 3 IHRC Inc., Atlanta, 30346 USA; 4 Weems Design Studio Inc., Suwanee, 30024 USA

**Keywords:** Dried blood spot, Pneumonia, Nasopharynx, Colonization, *Streptococcus pneumoniae*, *Haemophilus influenzae*

## Abstract

**Background:**

Dried blood spots (DBS) have been proposed as potentially tool for detecting invasive bacterial diseases.

**Methods:**

We evaluated the use of DBS for *S. pneumoniae* and *H. influenzae* detection among children in Mozambique. Blood for DBS and nasopharyngeal (NP) swabs were collected from children with pneumonia and healthy aged < 5 years. Bacterial detection and serotyping were performed by quantitative PCR (qPCR) (NP and DBS; *lyt*A gene for pneumococcus and *hpd* for *H. influenzae*) and culture (NP). Combined detection rates were compared between children with pneumonia and healthy.

**Results:**

Of 325 children enrolled, 205 had pneumonia and 120 were healthy. Pneumococci were detected in DBS from 20.5 and 64.2% of children with pneumonia and healthy, respectively; NP specimens were positive for pneumococcus in 80.0 and 80.8%, respectively. *H. influenzae* was detected in DBS from 22.9% of children with pneumonia and 59.2% of healthy; 81.4 and 81.5% of NP specimens were positive for *H. influenzae,* respectively.

**Conclusion:**

DBS detected pneumococcal and *H. influenzae* DNA in children with pneumonia and healthy. Healthy children were often DBS positive for both bacteria, suggesting that qPCR of DBS specimens does not differentiate disease from colonization and is therefore not a useful diagnostic tool for children.

## Background

Bacterial pneumonia is a leading cause of death in children worldwide, causing over 900,000 deaths annually in children aged < 5 years. The most common bacterial cause of pneumonia in children is *S. pneumoniae,* followed by *H. influenzae* type b (Hib) and *Staphylococcus aureus* [1, 2].

Since 1996, the Centro de Investigação em Saúde de Manhiça (CISM) has conducted surveillance for invasive bacterial diseases (IBD) among children in Mozambique rural area. Data from this surveillance system was instrumental for introducing the Hib vaccine in August 2009 [3] and 10-valent pneumococcal conjugate vaccine (PCV10) in April 2013. Invasive pneumococcal disease (IPD) incidence observed pre-PCV10 introduction was 245 cases per 100,000 among children < 5 years old, higher than what has been reported for this age group from other African sites, with an overall case fatality ratio of 14% [4].

Despite this high burden of IPD, the true incidence is likely underestimated given the challenges with IBD diagnosis. Diagnosis of IBD at CISM is currently made by culture; quantitative PCR (qPCR); and antigen tests (blood, cerebrospinal fluid or other sterile site fluids). Culture results can be highly influenced by prior antibiotic use, volume of specimen collected, specimen transport and storage conditions prior to processing, and culture media quality used to support the growth of fastidious bacteria like *S. pneumoniae* and *H. influenzae* [5]. To try to overcome these challenges, new potential diagnostic alternatives such as testing of dried blood spots (DBS) by qPCR have been proposed; yet they require validation before implementation [6–8].

DBS was first used for metabolic disorder screening in neonates, and its use was expanded to include pathogen detection [8]. DBS has been successfully used for detection of parasitic and viral diseases such as malaria, HIV, and dengue [9, 10]. Testing DBS by qPCR is an attractive alternative to conventional diagnostic methods because the cards are low cost, require minimal blood volume, can be stored at room temperature, it is easy to collect enough blood via finger-prick, and testing for pathogens relies on deoxyribonucleic/ribonucleic acid (DNA/RNA) detection which may be less influenced by antimicrobial therapy than culture [9, 10]. However, little is known about their use as a diagnostic tool for detecting bacteria among children with IBD, and even less is known about whether qPCR testing of DBS will detect pathogens that are part of the normal flora in the upper respiratory tract of healthy children [10–12].

The development and validation of a diagnostic test that is not greatly impacted by prior antimicrobial use and that is both sensitive and specific would lead to a better understanding of the IBD burden, particularly in low- and mid-income countries where laboratory capacity is often limited. We examined detection of *S. pneumoniae* and *H. influenzae* by qPCR using DBS and the prevalence of nasopharyngeal colonization with these pathogens among children with pneumonia and healthy children < 5 years from a rural area in Mozambique. For children with pneumonia who had undergone blood culture, we also compared those results with DBS findings.

## Methods

### Study area and population

The study was conducted between 2014 and 2015 among children aged < 5 years admitted to Manhiça District Hospital. Manhiça District is a rural area in southern Mozambique with a population of approximately 140,000 inhabitants. Since 1996, CISM has conducted IBD surveillance at this hospital, a 110-bed facility with 36 pediatric beds. Blood culture is routinely performed for all children aged < 2 years on admission and for children aged 2–14 years admitted with axillary temperature ≥ 38 °C. Bacterial isolation and detection is performed using standard methods in the hospital laboratory [3, 4].

In order to evaluate the value of DBS test for distinguishing patients with pneumonia and potentially bacteremia from those without pneumonia and assess the influence of colonization on test results, we enrolled children with pneumonia and healthy children < 5 years of age. Children with pneumonia were recruited at the hospital if they were hospitalized with severe pneumonia, while healthy children were randomly selected from the community using the Manhiça Demographic Surveillance System (DSS) [13]. Community workers visited household of selected healthy children for enrollment in the study. Severe pneumonia was defined as fever with cough or difficulty breathing associated with tachypnea and chest wall in-drawing [13]. Children recruited from the community were not enrolled if they had acute respiratory illness. The study protocol was approved by the Mozambique Ministry of Healthy and Centers for Disease Control and Prevention (CDC) Institutional Review Boards. Written informed consent was obtained from all parents or legal guardians prior to study enrollment. Demographic data were obtained for all participants.

### Specimen collection

Blood and nasopharyngeal (NP) swabs were collected from each child by trained staff. Blood for DBS was collected first, followed by NP swabs. For children with pneumonia, specimen collection was performed within 48 h of admission. Blood was collected through finger or heel prick, depending on the child’s age, and placed directly onto filter paper card containing 5 spots (Whatman Grade 903, cat#10535097). After the blood dried, the card was put inside the aluminum card package, transported at room temperature to Manhiça laboratory, stored at − 20 °C, as an extra precaution to preserve DNA integrity, and shipped to the CDC *Streptococcus* Laboratory for processing.

A single NP swab was collected using a flexible and sterile calcium alginate tipped applicator (Puritan® Calgiswab®, cat# 25–800 or 25–801). The swab was immediately placed in transport media containing 1.0 mL skim milk, tryptone, glucose, and glycerol (STGG-NP). Inoculated STGG-NP vials were kept at 4 °C within 4–5 h after collection and stored at -70C° until underwent culture and/or qPCR for *S. pneumoniae* and *H. influenzae*.

### Bacterial detection on DBS

All the five spots from each DBS paper filter were cut into four pieces (approximately 250 uL of blood), transferred to a round-bottomed tube, added 600 uL of ATL-buffer (Qiagen cat#1014758), and the tube vigorously vortexed for 10 s. The solution was incubated at 85 *°*C for 10 min, after added 20 uL of proteinase-K (600mAU/mL – Qiagen cat#19133), vortexed for 10 s, followed by incubation at 56 *°*C for 10 min. A volume of 600 uL of guanidinium-isothiocyanate-buffer (MagnaPure DNA Isolation Kit III - cat#03264785001) was added to the sample and incubated at room temperature for 5 min. The solution was transferred to the sample tube and DNA purification performed in the MagnaPure instrument set up for “DNA_Blood_external_lysis_V3_2”. The DNA elution volume was 100uL.

DNA was stored at − 20 °C until qPCR was performed using PerfeCTa® qPCR ToughMix® Low ROX™ (cat# 95114–012) for pneumococcal *lyt*A gene [14], *H. influenzae hpd* gene [15]. *hpd* positive samples were serotyped by qPCR [16], and *lyt*A positive samples were serotyped by quantitative multiplex PCR (qmPCR) assays covering the 37 most frequent pneumococcal serotypes (1, 2, 3, 4, 5, 6A/6B, 6C/6D, 7F/7A, 9 V/9A, 11A/11D, 12F/12A/12B/44/46, 14, 15F/15A, 16F, 18A/18B/18C/18D, 19A, 19F, 22F/22A, 23A, 23F, 33F/33C/37) [17].

### *S. pneumoniae* and *H. influenzae* detection from NP swab specimens

For pneumococcal isolation, a volume of 200 uL of STGG-NP was transferred to 5.0 mL Todd Hewitt broth containing 0.5% yeast extract and 1.0 mL of rabbit serum; the broth was incubated at 35–37 °C for 5 h and streaked onto blood agar plates for colony isolation [18]. Suspect colonies underwent optochin and bile solubility tests for pneumococcal identification. *S. pneumoniae* isolates were serotyped by Quellung reaction.

For *H. influenzae* isolation, a volume of 100 uL of the STGG-NP was transferred to a chocolate agar plate with bacitracin and the plate incubated overnight in 5% CO2 at 35–37 °C. Suspect colonies underwent Gram stain, oxidase, and X-V factor tests [19]. For species confirmation and serotyping, DNA was extracted from *H. influenzae* isolates and tested for *hpd* and serotyping genes by qPCR [15, 16].

For NP specimens, DNA extracts were obtained from 200 uL of STGG-NP using the protocol described above without the pretreatment step with ATL-buffer. DNA was stored at -20 °C until qPCR was performed for the pneumococcal *lyt*A gene [14], *H. influenzae hpd* gene [15], and serotyping as described above [16, 17].

We compared the proportion of specimens that were positive for pneumococcus or *H. influenzae* among children with pneumonia and healthy using Chi-square or Fisher’s Exact test when appropriate. Comparisons of DBS positivity among colonized and non-colonized children were also performed. *P*-values < 0.05 considered statistically significant.

## Results

Of 325 children enrolled, 205 had pneumonia and 120 were healthy controls from the community. Among the 203 and 119 children with pneumonia and healthy with age available, 134 (66.0%) and 19 (16.0%) were ≤ 1 year of age, respectively. Pneumococcal *lyt*A gene was detected from DBS in 20.5% (42/205) of children with pneumonia and in 64.2% (77/120) of healthy. The *H. influenzae hpd* gene was detected from DBS in 22.9% (47/205) of children with pneumonia and in 59.2% (71/120) of healthy. *lyt*A and *hpd* genes were detected simultaneously from DBS in 13.2 and 46.7% of children with pneumonia and heathy, respectively (Table 1).

**Table 1:** *S. pneumoniae* (*lyt*A) and *H. influenzae* (*hpd*) detection from DBS and NP swabs collected from children with pneumonia and healthy children from a rural area in Mozambique, 2015

	Children with pneumonia	Healthy children	***P*** value
	*N* = 205 n(%)	*N* = 120 n(%)	
**Dried Blood Spot PCR results**			
*lyt*A positive only	13(6.3)	21(17.5)	0.001
*hpd* positive only	18(8.8)	15(12.5)	0.11
*lyt*A *+ hpd* positive	29(14.2)	56(46.7)	< 0.001
Negative	145(70.7)	28(23.3)	< 0.001
**NP swab culture results**^**a**^			
*S. pneumoniae* only	43(20.9)	25(20.8)	0.9
*H. influenzae* only	14(6.8)	9(7.6)	0.8
*S. pneumoniae* + *H. influenzae*	102(49.8)	65(54.2)	0.4
Negative	45(22.0)	21(17.7)	0.35
**NP-swab qPCR results from culture negative specimens**			
	n/N (%)	n/N(%)	*P* value
*lyt*A positive	19/60(31.7)	7/30(23.3)	0.41
*hpd* positive	51/89(57.3)	23/46(50.0)	0.42

^a^ 1 (0.8%) NP swab was not processed for *H. influenzae* culture

**Fig. 1:**
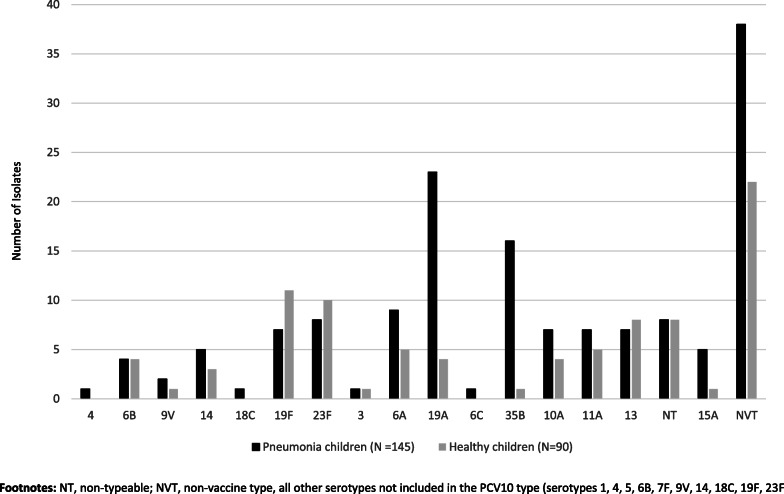
Pneumococcal serotype distribution from nasopharyngeal swabs for children with pneumonia and health children from Mozambique rural area, 2015

*S. pneumoniae* was detected in 80.0% (164/205) of children with pneumonia nasopharynx, 88.4% (145/164) of it was isolated by culture (Table 1). Among healthy children, 80.8% (97/120) were colonized with *S. pneumoniae*, 92.7% (90/97) of it was isolated by culture. Thirty-eight serotypes were identified amongst the pneumococcal isolates (Fig. 1). Five children with pneumonia were colonized with two pneumococcal serotypes. The *lyt*A gene was detected in 31.7% (19/60) and 23.3% (7/30) in NP swabs pneumococcal culture negative from children with pneumonia and healthy, respectively. Serotyping was performed on all 19 *lyt*A positive samples, but the serotype/serogroup was determined only for seven (3, 6A/6B, 18, 19A, 19F, 20, 23F). In children with pneumoniae 19.3% of *S. pneumoniae* isolates serotypes were included in the PCV10, (serotype 23F, *n* = 8; 19F, *n* = 7; 14, *n* = 5; 6B, *n* = 4; 9 V, *n* = 2; 4, *n* = 1; 18C, n = 1) and in healthy 32.2% (serotype 23F, *n* = 10; 19F, *n* = 11; 14, *n* = 3; 6B, n = 4; 9 V, n = 1).

*H. influenzae* was detected in the nasopharynx of 81.4% (167/205) of children with pneumonia, (116/167 69.4%) isolated by culture (Table 1), most were non-typeable (NT) (*n* = 112), and four were serotyped (serotype c, *n* = 1; e, *n* = 3). Among healthy children, 81.5% (97/119) were colonized with *H. influenzae* in the nasopharynx; 76.2% (74/97) isolated by culture, most were NT (*n* = 66) and eight were serotype (serotype a, n = 3; b, n = 3; c, *n* = 1; d, n = 1). The *hpd* gene was detected in 57.3% (51/89) NP swabs *H. influenzae* culture negative from children with pneumonia (NT, *n* = 45; serotype a, n = 1; b, *n* = 2; e, n = 2; f, n = 1) and in 51.1% (23/45) among healthy children (NT, *n* = 16; serotype a, n = 2; b, n = 1; c, n = 1; e, n = 2; f, n = 2). Co-colonization with *S. pneumoniae* and *H. influenzae* was detected in the nasopharynx of 111 (54.1%) children with pneumonia and 69 (57.9%) healthy.

The combination of pneumococcal colonization and detection of *lyt*A in the DBS was found in 92.8% (39/42) of children with pneumonia, 85.7% (36/42) of the *S. pneumoniae* were isolated from the nasopharynx. While for healthy children this combination was found in 89.6% (69/77); 85.7% (66/77) of *S. pneumoniae* isolated by culture. The combination of *H. influenzae* colonization and *hpd* detection in DBS from children with pneumonia was found in 93.6% (44/47), for which 70.2% (33/47) of the *H. influenzae* were isolated from the nasopharynx; for healthy children it was 92.9% (66/71), 76% (54/71) of *H. influenzae* isolated by culture (Table 1). DBS was more likely to be positive for pneumococci or *H. influenzae* if the subject was colonized, in both groups (Table 2).

**Table 2:** Results of bacterial detection from DBS specimens by NP colonization status among children with pneumonia and healthy children

	**With Pneumococcal colonization**			**Without Pneumococcal colonization**			***P*value**
	Total	DBS positive	% DBS positive	Total	DBS Positive	% DBS positive	
Children with pneumonia	164	39	23.8	41	3	7.3	0.02
Healthy children	97	69	71.1	23	8	34.8	0.001
	**With*H. influenzae*colonization**			**Without*H. influenzae*colonization**			***P*value**
	Total	DBS Positive	% DBS positive	Total	DBS Positive	% DBS positive	
Children with pneumonia	167	44	26.3	38	3	7.9	0.02
Healthy children	97	66	68.0	23	5	21.7	< 0.001

Only DBS *lyt*A positive specimens with DNA yield concentrations with cycle threshold value (Ct) < 32 were able to be serotyped. Fifteen DBS *lyt*A positive from children with pneumonia were serotyped; of these, 66.6% (10/15) of the serotypes matched pneumococcal serotypes isolated in the nasopharynx. In healthy children, 30 DBS *lyt*A positive were serotyped, and 33.3% (10/30) were the same serotype/serogroup as was found in the nasopharynx. The pneumococcal serotype isolated in the nasopharynx of 16 children (7C, 10A, 13, 15B, 15C, 17F, 23B, 24F, 28F, 34, 35B, and 38) could not be compared with the DBS positive specimens because they are serotypes not included in the qmPCR assays.

Blood culture results were available for 185/205 (90.2%) children with pneumonia: *S. pneumoniae* [6], *Staphylococcus aureus* [2], *H. influenzae* [1]*, Salmonella sp* [1]*, Escherichia coli* [1]*,* and 174 were culture negative. All blood culture positive for *S. pneumoniae* were from children < 1 year old. The DBS and NP swabs from these six children were also positive for pneumococcus (100% sensitivity). In three children, the same pneumococcal serotype was identified in the blood culture, DBS, and nasopharynx (serotype 6A [*n* = 2] and serotype 15A). We could not identify and compare the DBS pneumococcal serotype from the other three children because the *S. pneumoniae* isolated from the blood and nasopharynx were serotypes not encompassed in the qmPCR (10A, 13, 35B).

*H. influenzae* b was isolated in only one blood culture from a child > 1 year old, and the same bacteria was also detected in the DBS and nasopharynx (100% sensitivity). There were no differences in the proportion of children with pneumonia or healthy children who had a DBS positive test result or had pneumococcal or *H. influenzae* nasopharyngeal colonization by age group (Table 3).

**Table 3:** Proportion of DBS and NP specimens with *S. pneumoniae* and *H. influenzae* detected by age group* among children with pneumonia and healthy children

	Age **<** 1 year			Age > 1 to 5 years			***P*** value
	DBS positive	Total	%	DBS positive	Total	%	
Children with pneumonia	42	134	31.3	24	69	34.8	0.62
Healthy children	14	19	73.7	80	100	80.0	0.53
	NP swab *S.pneumoniae* positive	Total	%	NP swab *S.pneumoniae* positive	Total	%	
Children with pneumonia	112	134	83.6	51	69	73.9	0.10
Healthy children	16	19	84.2	81	100	81.0	0.74
	NP swab *H.influenzae* positive	Total	%	NP swab *H.influenzae* positive	Total	%	
Children with pneumonia	108	134	80.6	58	69	84.0	0.54
Healthy children	17	19	89.5	80	100	80.0	0.33

*age missing for 2 children with pneumonia and 1 healthy child

## Discussion

Our evaluation indicated that qPCR of blood from DBS specimens is not a useful method for distinguishing a bacterial pneumonia from bacterial colonization in children. DBS specimens were more likely to be positive for *S. pneumoniae* and/or *H. influenzae* when a child was colonized by these bacteria in the nasopharynx. We detected more pneumococcal and *H. influenzae* DNA in healthy children’s DBS than in DBS samples from children with pneumonia, and co-detection of *S. pneumoniae* (*lyt*A) and *H.influenzae* (*hpd*) genes was found in almost half the DBS samples from healthy children. Bacterial DNA detection in blood from healthy children could be related to transient bacteremia or DNAemia from bacterial fragments in the bloodstream. Concerns have already been reported that positive pneumococcal detection in blood by qPCR may not reflect pneumococcal disease [12, 20, 21]. The usefulness of pneumococcal qPCR on blood was reported as limited in diagnosing childhood pneumococcal pneumonia by the Pneumonia Etiology Research for Child Health (PERCH) since positivity among controls (5.5%) was comparable to that in pneumoniae cases not confirmed for any bacterial pathogens (6.3%), and among cases confirmed for nonpneumococcal bacteria (11.2%) [21].

Limited data are available for bacterial detection using DBS and many studies have not included healthy controls when evaluating the performance of DBS for detection of pneumococcus or *H. influenzae*. A study with Nigerian children only found 0.96% positivity for *S. pneumoniae* on DBS of 1038 febrile children and 1.3% *S. pneumoniae* positive from 79 healthy children [22].

Before DBS collection began, we performed tests to optimize methods using serial dilution of human blood spiked with *S. pneumoniae*, *S. agalactiae* and *S. pyogenes* isolates from several serotypes to evaluate the Whatman Grade 903 (cat#10535097), and the FTA Elute Micro CardTM (cat#WB120401) (data not shown). Several protocols for DBS DNA extraction were also tested [11, 23, 24], including some automated extraction systems (Nuclisens EasyMag and MagNAPure). Only after extensive testing of these protocols a final protocol with the most optimal results was established for this study (data not shown).

Each DBS spot has a 12 mm diameter, corresponding to approximately 50 uL of blood. Previously reported protocols had DNA extracted from just one DBS spot (50 uL) or one 3 mm punch containing around 12 uL of blood [11, 12, 23–25]. The relatively low volume of blood extracted, and DNA added into the qPCR reaction is likely to be the major factor for the lack of sensitivity and inconsistent bacterial detection in previous DBS testing. The improved DNA extraction protocol allowed for use of all five (250 uL) blood spots on each card. This increase yielded higher availability of purified bacterial DNA, which in association with a more stable Taq DNA polymerase that better resisted common residual qPCR inhibitors from blood, allowed for better performance of the amplification reaction. The methods applied here enhanced DBS qPCR testing for *S. pneumoniae* detection from 2 to 9% positive (1 spot from the DBS card) in previous reports [12, 26] to 20.5% (5 spots) when testing symptomatic children. While these methods were not helpful for diagnosis of disease caused by pneumococci or *H. influenzae*, the technique might be useful for detection of systemic infections caused by bacteria that are not commonly carried in the upper respiratory tract.

Pneumococcal nasopharyngeal carriage prevalence, considering the detection by culture and qPCR, was high for children with pneumonia (80%) and for those who were healthy (80.8%), like what has been previously reported in cross-sectional pneumococcal carriage surveys conducted in the same rural area in Mozambique [27, 28].

Children who were colonized with *S. pneumoniae* or *H. influenzae* were at least 2-fold more likely to have a DBS positive for the pathogen they were carrying compared to those who were not colonized.

Our results agree with Morpeth et al. [21] that reported higher blood pneumococcal qPCR positivity (almost 2-fold) among those controls with nasopharyngeal carriage. The impact of colonization on DBS test results limits the specificity of DBS for detecting disease, particularly in areas where colonization is common. The high bacterial load in the nasopharynx often found in young children could have an impact on the amount of DNA in the child’s bloodstream and urine. Similarly, tests for detecting pneumococcal antigen in urine were significantly more likely to be positive among children who were nasopharyngeal carriers of pneumococci than for those who were not [29]. The increased likelihood of qPCR detection in blood samples when testing children with densely colonized nasopharynx was also reported [30]. The accuracy of pneumococcal detection by qPCR in serum has also been evaluated for children who had pneumococci detected by culture of cerebrospinal fluid and blood; qPCR detected pneumococci in serum from these children, but serum was also positive in 17% of healthy controls [20].

The use of DBS for of *S. pneumoniae* and *H. influenzae* detection was previously evaluated on children hospitalized with pneumonia from Mozambique and Morocco [26]. Even though our study was conducted in the same District in Mozambique, our results differ. The detection rates for pneumococci and *H. influenzae* among children with pneumonia were 9.0 and 3.3% in the earlier study compared to 20.5 and 22.9% in our study, respectively. Among healthy children, these authors [26] found only 1.9% were DBS positive for pneumococci compared to 64.2% in ours. They also could not associate pneumococci isolated from blood culture (3.1%) with the corresponding DBS, whereas in our study the same serotypes of *S. pneumoniae* and *H. influenzae* found on blood culture (3.2%) were simultaneously identified in the DBS and NP swabs of children with pneumonia. A few factors could explain the differences: 1) our results are based on a comprehensive population study with paired nasopharyngeal and DBS results from children with pneumonia and healthy; 2) all children enrolled in our analysis were < 5 years old, but Selva et al. [26] enrolled children up to 10 years old (older children have lower colonization rates); 3) we optimized DNA extraction procedure that allowed all five DBS spots from each card to be included, resulting in approximately 250 uL of blood volume extracted but Selva et al. [26] used 100 uL; 4) we used a different nucleic acid purification system; and 5) we also used a Taq DNA polymerase that is more resistant to qPCR inhibitors from blood.

Our study has several limitations. First, we restricted enrollment of children with pneumonia to one hospital, Manhiça District Hospital, which is the main referral hospital for the District from where healthy children were recruited. As a referral hospital, it admits many patients after they have been treatment elsewhere. We also could not assess the time of administration of inpatient antimicrobial use in relation to the collection of DBS. Some enrolled children likely received the intravenous antibiotics prior to collection of DBS. Collection of specimens from pneumonia children after they received antibiotics, including any antibiotics received before admission as well, may have reduced detection of pneumococcal DNA from their specimens. A cross-sectional survey conducted in the same period and area of Mozambique found 97.4% of children with pneumonia had antibiotics recently compared to 27% among children without pneumonia [27]. The vaccination status of the children was obtained, and Mozambique has high vaccine coverage [28]. However, we could not assess the date of the last PCV10 dose compared to the swab or blood collection. Manhiça has a high pneumococcal colonization rate, similar to other countries in Africa, but different from developed countries [28, 31]. DBS may perform better in settings where pneumococcal colonization is not as prevalent as in Mozambique.

Another limitation is that in several instances we could not determine the serotype from *lyt*A positive DBS samples, either because the DNA concentration recovered was too low (Ct > 32) or the serotypes were not encompassed in the qmPCR assays.

## Conclusions

We found that DBS positive results were highly associated with pneumococcal and *H. influenzae* nasopharyngeal colonization, suggesting that qPCR testing for DBS samples did not distinguish colonization from invasive disease and therefore is not likely to be useful for diagnosis of pneumonia etiology in children.

## Data Availability

The data generated or analyzed during this study are included in this published article.

## Abbreviations

CDC: Centers for Disease Control and Prevention
CISM: Centro de Investigação em Saúde de Manhiça
DBS: Dried Blood Spots
DSS: Demographic Surveillance System
IBD: invasive bacterial diseases
IPD: invasive pneumococcal disease
NP: nasopharyngeal
NT: non-typeable
PERCH: Pneumonia Etiology Research for Child Health
PCV10: 10-valent pneumococcal conjugate vaccine
qmPCR: quantitative multiplex PCR
qPCR: quantitative PCR
STGG: skim milk, tryptone, glucose, and glycerol
STGG-NP: skim milk, tryptone, glucose, glycerol inoculated with nasopharyngeal swab
